# Large-scale inference of gene function through phylogenetic annotation of Gene Ontology terms: case study of the apoptosis and autophagy cellular processes

**DOI:** 10.1093/database/baw155

**Published:** 2016-12-26

**Authors:** Marc Feuermann, Pascale Gaudet, Huaiyu Mi, Suzanna E. Lewis, Paul D. Thomas

**Affiliations:** 1 Swiss-Prot group; 2 CALIPHO group, SIB Swiss Institute of Bioinformatics, Centre Medical Universitaire, Switzerland 1 rue Michel Servet, 1211 Geneva 4, Switzerland; 3 Division of Bioinformatics, Department of Preventive Medicine, Keck School of Medicine of USC, University of Southern California, Los Angeles, CA, USA and; 4 Lawrence Berkeley National Laboratory, Genomics Division, Berkeley, CA, USA

## Abstract

We previously reported a paradigm for large-scale phylogenomic analysis of gene families that takes advantage of the large corpus of experimentally supported Gene Ontology (GO) annotations. This ‘GO Phylogenetic Annotation’ approach integrates GO annotations from evolutionarily related genes across ∼100 different organisms in the context of a gene family tree, in which curators build an explicit model of the evolution of gene functions. GO Phylogenetic Annotation models the gain and loss of functions in a gene family tree, which is used to infer the functions of uncharacterized (or incompletely characterized) gene products, even for human proteins that are relatively well studied. Here, we report our results from applying this paradigm to two well-characterized cellular processes, apoptosis and autophagy. This revealed several important observations with respect to GO annotations and how they can be used for function inference. Notably, we applied only a small fraction of the experimentally supported GO annotations to infer function in other family members. The majority of other annotations describe indirect effects, phenotypes or results from high throughput experiments. In addition, we show here how feedback from phylogenetic annotation leads to significant improvements in the PANTHER trees, the GO annotations and GO itself. Thus GO phylogenetic annotation both increases the quantity and improves the accuracy of the GO annotations provided to the research community. We expect these phylogenetically based annotations to be of broad use in gene enrichment analysis as well as other applications of GO annotations.

**Database URL:** http://amigo.geneontology.org/amigo

## Introduction

The Gene Ontology (GO) is an international effort between multiple groups aimed at describing the functions of gene products in a uniform manner (1). To date, over 40 000 terms have been created, that define proteins' biochemical activities [10 000 Molecular Function terms (MF)], their biological roles [27 000 Biological Process terms (BP)] as well as the sub-cellular localization in which they act [3700 Cellular Component terms (CC)]. Manual GO annotation is the result of summarizing experimental results from peer reviewed scientific papers.

Since most gene products have not been experimentally characterized, accurate methods for gene function prediction, whether manual or automatic, are needed to complete the annotation of partially characterized organisms, as well as to annotate new genomes. The most commonly used manual approach is by sequence similarity search using tools such as BLAST (2). Another manual approach takes into account the genetic context, such as operons, to infer the function of neighboring genes (3). This method is usually limited to prokaryotes.

Automated methods for functional prediction include HAMAP, High-quality Automated and Manual Annotation of Proteins, developed by the Swiss-Prot group of the SIB Swiss Institute of Bioinformatics. In HAMAP, manually curated family profiles are associated with annotation rules that trigger automatic annotation of proteins belonging to these well-conserved families or subfamilies (http://hamap.expasy.org/) (4). Another automatic annotation approach is InterPro2GO (5), in which GO terms are automatically assigned based on the presence of a protein domain. The domains are manually assigned putative functions, and these functions are propagated automatically onto proteins containing those specific domains. Yet another strategy is to use the evolutionary relations between sequences to predict function based on the known roles of members of the same phylogenetic group, for example the EnsemblCompara-GeneTrees method (http://www.ensembl.org/index.html) (6). Compara computes orthologs from the gene trees, and uses automatic rules for ‘propagating’ experimental annotations among closely related orthologs in, separately, either vertebrates or plants. The limitations for all of the automatic methods are two-fold. First is the issue of systematic errors, for example incorrectly associating a function with a particular protein motif when in fact the connection only applies in a subset of cases, or the occasional rule that is missing or incorrect. Corrections for these systematic inaccuracies are commonly brought to light sporadically when manually reviewed. The second, perhaps more consequential issue, is that the automated methods are generally quite conservative in the inferences being made to avoid over specifying the biology.

The Phylogenetic Annotation and INference Tool (PAINT) developed by the Gene Ontology Consortium, integrates phylogenetic trees, multiple sequence alignments, experimental GO annotations, as well as literature references pointing to the original data. Like Compara, PAINT also uses a tree-based approach, but with one crucial difference (7). PAINT annotations are done manually, with curators reviewing all available data to construct an explicit evolutionary model. This construction process includes selectively choosing which particular annotations can be propagated based on other available, relevant evidence, such as the conservation of important residues and the conservation of processes across different taxonomic ranges. This manual process enables the curator to make decisions based on factors beyond the phylogenetic relatedness of sequences.

To demonstrate the usefulness of PAINT in providing a large number of high confidence annotations and a coherent annotation corpus across representative species, we annotated the families of those proteins involved in two well conserved, well characterized processes: apoptosis and autophagy. These two cellular processes control the fate of the cell, leading to survival (autophagy) or death (apoptosis) in response to stresses such as starvation or DNA damage. The work presented here focuses specifically on the execution phase for apoptosis, and, in the case of autophagy, the autophagosome assembly. These steps are clearly defined by GO and bounded by precise starts and ends. The fact that these processes are well characterized provides a large number of primary experimentally based annotations, which is essential for comprehensive annotation in PAINT. Moreover, both processes are evolutionarily quite ancient, which allows inference of protein function across large phylogenetic distances.

## Results and discussion

### Process-by-process versus paper-by-paper approach for phylogenetic annotation

The approach we present focuses on the annotation of specific processes. The aim of this strategy is to provide a coherent set of annotations for all genes known to be involved in a particular process, rather than to annotate individual findings from different papers in isolation. The phylogenetic annotations are done using PAINT. To illustrate our process-based phylogenetic annotation approach, we show our results from the annotation of two well-characterized processes, apoptosis and autophagy.

### Annotation tool

The GO Phylogenetic Annotation Project applies PAINT to annotate PANTHER families (8), each containing between 5 and 5000 proteins. The number of genomes included increases regularly, keeping pace with the Quest for Orthologs's group dataset (http://www.ebi.ac.uk/reference_proteomes/) (9). The PAINT tool visualizes speciation, duplication and horizontal gene transfer events, sequence alignments and descriptive data and external links for both proteins and annotations. PAINT annotation is a two-step process. In the *first step*, curators create a model of evolution that is consistent with the observed experimental annotations of modern-day sequences. The curator infers the most likely point in evolution at which a GO function first evolved, taking into account the distribution of that function among experimentally characterized genes. Once constructed, this model is used in a *second step* to create inferred annotations over the entire tree, with each function being inherited from the ancestor(s) in which it first evolved, as determined in step 1. Curators may also identify points in evolution where a function was likely lost. These functional loss events may be supported by sequence evidence (the loss of an active site, a binding site or a domain critical for a particular function), or by an accelerated evolutionary rate leading to low sequence conservation. The support for each inferred annotation is explicitly captured: annotations to ancestral genes are linked to the identifiers of the sequences having experimental data and annotations to descendant genes refer back to their ancestral genes.

### Case study I: apoptosis execution phase

**Figure 1.**
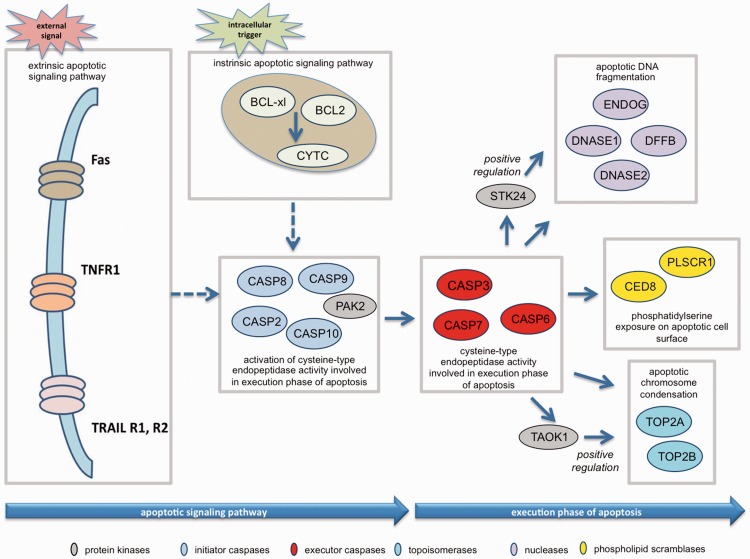
Representation of apoptosis in the Gene Ontology. The main steps (or GO processes) are indicated in each box, and the proteins mediating these processes are shown: Initiator caspases (pale blue), executor caspases (red), topoisomerases (turquoise), protein kinases (grey), phospholipid scramblases (yellow), and nucleases (purple).

Apoptosis is a program by which cells are eliminated from the body under specific and highly regulated conditions (10, 11). In GO, apoptosis (GO:0006915) is defined as ‘A programmed cell death process which begins when a cell receives an internal (e.g. DNA damage) or external signal (e.g. an extracellular death ligand), and proceeds through a series of biochemical events (signaling pathway phase) which trigger an execution phase. When the execution phase is completed, the cell has died’. This apoptotic process is thus divided into ‘apoptotic signaling pathway’ (GO:0097190) and ‘execution phase of apoptosis’ (GO:0097194) (Figure 1). Once initiator caspases are activated, execution of apoptosis (GO:0097194) takes place as follows: ‘The execution phase is the last step of an apoptotic process, and is typically characterized by rounding-up of the cell, retraction of pseudopodes, reduction of cellular volume (pyknosis), chromatin condensation, nuclear fragmentation (karyorrhexis), plasma membrane blebbing and fragmentation of the cell into apoptotic bodies’. The execution phase of apoptosis is further divided into the following steps in GO: ‘cellular component disassembly involved in execution phase of apoptosis’ (GO:0006921), ‘cysteine-type endopeptidase activity involved in execution phase of apoptosis’ (GO:0097200) and ‘phosphatidylserine exposure on apoptotic cell surface’ (GO:0070782).

The regulation of apoptosis is complex (10). Distinguishing direct versus indirect roles in the execution of apoptosis can be challenging, as it often necessitates additional knowledge to understand the full biological context of the data presented in research articles. Therefore, we decided to focus our analysis on proteins for which there is evidence of direct participation in the execution phase of apoptosis.

#### Phylogenetic annotation of apoptosis-related families predicts many new annotations, even for human genes

The term ‘execution phase of apoptosis’ (GO:0097194) and its children have 114 experimental GO annotations for 77 proteins in the Gene Ontology database (15 April 2015) with members in 34 different PANTHER families. From these primary annotations, 12 families were annotated using PAINT as participants in the execution of apoptosis (Table 1). The other 22 families were not annotated because the evidence for a role in the execution of apoptosis was too weak to predict that the function was conserved. The 12 annotated families contained a total of 2897 proteins, 289 or roughly 10% of them having at least one GO annotation based on experimental data (related to a function, a process or a cell component). The total number of all experimentally based annotations from these 289 proteins was 3964. Phylogenetic annotation from those descendent proteins led to 111 annotations on ancestral nodes (flagged with the evidence code ‘inferred from biological descendant’, IBD) resulting in 16 639 annotations to descendants (‘inferred from biological ancestor’, IBA), including 300 new annotations for 67 human proteins. Specifically for the ‘execution phase of apoptosis’ process, 172 descendent proteins were annotated as participating.

**Table 1.** Annotation statistics for proteins with members participating in the execution of apoptosis

Protein families^a^			Experimental annotations^b^			Tree annotations^c^				Functions^d^
PTHR family ID	Representative human proteins	Protein count	Proteins	EXP	Terms	Annotated Proteins	IBD	IBA	Terms	
PTHR13067	DFFB	22	3	16	10	19	3	53	3	Catalytic subunit of DNA fragmentation factor
PTHR11371	DNASE1	93	8	40	17	92	4	268	4	Deoxyribonuclease
PTHR10858	DNASE2	95	7	25	11	94	3	276	3	Deoxyribonuclease
PTHR13966	ENDOG	116	9	53	27	112	5	332	4	Endonuclease-related protein
PTHR23248	PLSCR1	195	13	135	45	193	5	576	5	Phospholipid scramblase
PTHR10169	TOP2A, TOP2B	212	24	267	108	211	12	1,658	12	DNA topoisomerase/gyrase; participates in chromosome condensation
PTHR10454	Caspases	418	50	1135	255	390	23	1576	22	Cysteinyl-aspartate-cleaving protease
PTHR31773	Metacaspases (MCA1 in fungi, MC1 in plants)	106	5	19	11	67	5	328	5	Cysteinyl proteases
PTHR31810	Metacaspases (MC4-MC9 in plants)	44	4	28	17	16	3	49	3	Cysteinyl proteases
PTHR24361	STK24, TAOK1, PAK2	1441	163	2 227	539	1441	44	11 407	34	Ser/Thr kinases (PAKs and MAPKs) STE family (yeast STE20-related)
PTHR32129	XKR8	112	2	7	3	16	2	32	2	Phospholipid scramblase
PTHR16024	Ced-8 (*C. elegans*)	43	1	12	7	42	2	84	2	Phospholipid scramblase
Total		2897	289	3964	1050	2693	111	16 639	99	

a The Protein families section defines the protein families annotated for apoptosis, including: (i) the PANTHER family ID; (ii) a representative protein, human whenever available or a well-characterized member of the family, with the species or phyla in parenthesis; (iii) the number of proteins in the family.

b The Experimental annotations section summarizes the experimental annotations available for PAINT inference. For each family: (i) the number of proteins (having at least one experimental annotation; (ii) the total number of different experimental annotations; (iii) the number of distinct GO terms associated with family members.

c The Tree annotations section summarizes the annotations inferred using PAINT. For each family: (i) the total number of proteins having at least one inferred annotation; (ii) the number of distinct IBD annotations (Inferred from Biological Descendant), representing the point in evolution in which the inferred function first evolved; (iii) the number of IBA annotations (Inferred from Biological Ancestor); inherited from the IBD annotations of the tree nodes from which the sequence has evolved; (iv) the number of distinct GO terms used for inference. Differences with the second tree annotation column are due to using more specific terms.

d The Function section gives the major function of the family.

### Case study II: autophagosome assembly

Autophagy is a highly conserved lysosome-dependent degradation process involved in the basal turnover of proteins and organelles through the degradation and recycling of cellular components (12). Contrary to apoptosis, autophagy is an adaptive response to stress that promotes survival (13). Autophagy plays an important role in the response to starvation (14) and in the defense response against intracellular pathogens (15). It has also been proposed that autophagy can lead to cell death and morbidity and act as an alternative to apoptosis (16). Our understanding of the molecular mechanisms mediating autophagy results from genetic studies in yeast, in which 35 autophagy-related (ATG) genes have been identified. Most of them are well conserved across eukaryotes and are essential for the formation and expansion of autophagosomes (17, 18).

Three types of autophagy have been described: chaperone-mediated autophagy, microautophagy and macroautophagy. Chaperone-mediated autophagy (CMA) specifically degrades proteins that contain a CMA-targeting motif recognized by a cytosolic chaperone that targets them to the lysosome. Microautophagy occurs through the direct engulfment of cytoplasmic materials by the lysosome. Finally, macroautophagy is the engulfment of cytosolic material and organelles by double-membrane vesicles called autophagosomes (19). Depending on the cellular component targeted for recycling, the macroautophagy process is known as mitophagy for mitochondria degradation (20, 21), nucleophagy for nuclei degradation (22), pexophagy for peroxisomes breakdown (12, 23), ribophagy for ribosomes (24), reticulophagy for endoplasmic reticulum (25, 26), lipophagy for lipids (27, 28), glycophagy for glycogen (29) and aggrephagy in the case of aggregates of proteins (30). In addition to its original meaning of self-eating, autophagy can also degrade invading pathogens such as bacteria. This type of autophagy is known as xenophagy (15).

**Figure 2.**
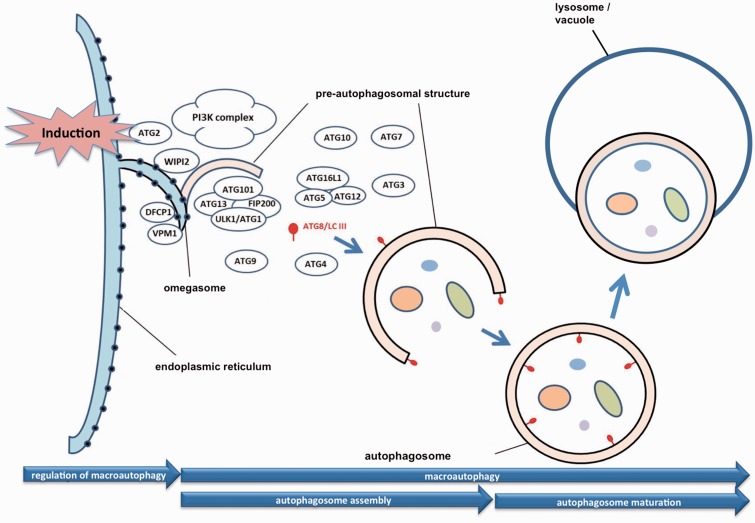
Representation of macroautophagy in the Gene Ontology. Induction of autophagy occurs in responses to stresses such as by starvation, and can be mediated by pathways such as the TOR pathway. The autophagosome begins from an extension of the endoplasmic reticulum membrane, and eventually fuses with the vacuole, leading to the degradation of its contents.

There are several more specific terms under the GO term ‘autophagy’ (GO:0006914) describing the different autophagic sub-processes described above (Figure 2). We focused our analysis on one clearly defined step in macroautophagy, the autophagosome assembly (GO:0000045). The degradation of the contents of the autophagosome by the lysosome that occurs after autophagosome assembly was considered outside the scope of the present analysis. Autophagosome formation is dynamically regulated by starvation and other stresses and involves complex membrane reorganization. The early steps of autophagosome formation have only recently been characterized (31). Autophagic signals induce the formation of omegasomes, membranous structures derived from the endoplasmic reticulum (ER), probably at ER-mitochondria contact sites. Omegasomes serve as intermediates for genesis of the isolation membrane, also called pre-autophagosomal structure or phagophore, which becomes a double membrane vesicle, the autophagosome (31–33). However, this is still a simplified picture of the process since recent studies also showed that contribution from other organelles and membranes such as vesicles derived from the Golgi, plasma membrane and endosomes also contribute to autophagosome expansion (31). Autophagosomes eventually fuse with lysosomes to deliver their content (cytosolic fractions or organelles) for degradation and recycling.

It is often challenging to distinguish the different structures and steps of autophagosome formation in the literature. Most of the proteins involved in autophagosome assembly have been localized to cytoplasmic and perinuclear punctate structures, probably corresponding to early steps of autophagosome assembly, as well as to perivacuolar punctate structures that could represent late steps of the autophagosome assembly. Moreover, the poor resolution of the classical protein localization techniques often precludes the discrimination of the different autophagosome formation steps.

#### Phylogenetic annotation of autophagy-related families predicts many new annotations, even for human genes

At the time of annotation, the GO database contained 98 experimental annotations to ‘autophagosome assembly’ (GO:0000045) for 85 proteins, corresponding to 30 PANTHER families. From these data, we have propagated this term to 508 proteins belonging to 24 families. These families include ER proteins, most of the core autophagy proteins (ATGs), as well as SNARE and RAB proteins, key regulators of the membrane trafficking and fusion (31). The 24 families annotated to autophagosome assembly contain a total of 6453 proteins, 559 of them having at least one experimentally supported GO annotation (either a function, a process, and/or a cell component) for a total of 4981 GO annotations (Table 2). Phylogenetic annotation of those terms led to 385 IBD annotations and 52 650 IBA annotations, including 923 new annotations to 157 human proteins.

**Table 2.** Annotation statistics for proteins with members participating in autophagosome assembly

Protein families^a^			Experimental annotations^b^			Tree annotations^c^				Function^d^
PTHR family ID	Representative human proteins	Protein count	Proteins	EXP	Terms	Annotated Proteins	IBD	IBA	Terms	
PTHR13430	ATG13	70	4	20	10	29	16	166	6	ULK1 kinase activator
PTHR23160	ATG11 (*S. cerevisiae*)	23	7	23	10	20	2	39	2	Scaffold protein
PTHR13664	ATG14	24	3	16	10	23	3	69	3	Membrane recruitment component of the PI3-kinase complex
PTHR14957	ATG10	30	2	10	8	30	5	145	5	E2 for ATG12-ATG5 conjugation
PTHR31671	TP53INP2	37	4	35	21	36	4	140	4	Scaffold protein that recruits ATG8 family proteins
PTHR13222	RB1CC1	44	5	30	23	44	6	257	6	Autophagosome formation
PTHR13292	ATG101	49	4	16	13	47	2	95	2	Protects ATG13 from proteasomal degradation
PTHR13385	ATG12	49	9	40	23	49	8	383	8	Ubiquitin-like protein
PTHR13040	ATG5	55	10	75	44	55	8	430	8	Component of E3 ubiquitin ligase for ATG8-PI conjugation
PTHR13038	ATG9	70	12	41	39	70	8	549	8	Bridging protein
PTHR19878	ATG16L1, ATG16L2	70	7	22	14	70	3	330	3	Stabilizes the ATG5-ATG12 conjugate
PTHR12768	BECN1	74	9	134	59	74	11	744	11	Regulatory component of the PI3-kinase complex
PTHR12866	ATG3	74	8	41	22	74	6	431	6	E2 for ATG8-PI conjugation
PTHR13190	ATG2A, ATG2B	81	6	27	18	81	6	479	6	Localizes ATG18 to omegasome and PAS
PTHR24348	ULK1, ULK2, ULK3	127	14	203	91	127	12	1052	12	Kinase for ATG9
PTHR22624	ATG4A	137	9	52	27	137	10	1356	10	ATG8 protease
PTHR11227	WIPI1, WIPI2	199	14	135	65	199	12	2174	12	PI(3,5)P2 regulatory complex
PTHR10969	MAP1LC3, GARABAP, …	213	26	239	70	213	14	2148	12	Membrane fusion, autophagosome assembly
PTHR10281	VMP1	223	22	91	49	222	12	853	10	Early step in autophagosome assembly
PTHR10048	PIK3C3	420	63	758	288	420	42	4488	36	Catalytic component of the PI3-kinase complex
PTHR10555	SNX4 (Sorting nexins)	521	57	546	129	520	14	2820	14	Sorting nexin involved in autophagosome assembly
PTHR19957	STX17 (syntaxins)	528	73	681	233	525	18	4681	18	SNARE of the autophagosome
PTHR10953	ATG7	631	64	423	159	627	51	3309	43	E1-like activating enzyme
PTHR24073	RAB1A, RAB23	2753	129	1321	307	2748	117	25 459	68	Rab GTPase involved in membrane recognition and fusion
Total		6453	559	4982	1740	6432	385	52 650	310	

a The Protein families section defines the protein families annotated for autophagy, including: (i) the PANTHER family ID; (ii) a representative protein, human whenever available, or a well-characterized member of the family, with the species or phyla in parenthesis; (iii) the number of proteins in the family.

b The Experimental annotations section summarizes the experimental annotations available for PAINT inference. For each family: (i) the number of proteins having at least one experimental annotation; (ii) the number of different experimental annotations; (iii) the number of distinct GO terms associated with family members.

c The Tree annotations section summarizes the annotations inferred using PAINT. For each family: (i) the number of proteins having at least one inferred annotation; (ii) the number of IBD annotations (Inferred from Biological Descendant), representing the point in evolution in which the inferred function first evolved; (iii) the number of IBA annotations (Inferred from Biological Ancestor); inherited from the IBD annotations of the tree nodes from which the sequence has evolved; (iv) the number of distinct GO terms used for inference.

d The Function section gives the major function of the family.

The 24 families are among the 42 families having primary GO annotations to autophagy-related GO terms. The proteins in the 18 other families are involved in events upstream and downstream of the autophagosome vacuole assembly, and so are not presented in our results. These include for example, PTHR21493, that contains the *S. cerevisiae* Atg15p lipase, required for in the degradation of membranes targeted inside the vacuole (34, 35), and the vacuolar transporter mediating the efflux of amino acids resulting from autophagic degradation, belonging to PTHR11360 (36).

Interestingly, after the families were annotated, new annotations were added to some of the proteins that are consistent with the inferences we made. For instance, 13 proteins for which we had inferred ‘autophagy’ have since been linked to this term by experimental evidence, including the human protein ATG4B. For the more precise term ‘autophagosome assembly’, five additional new experimental annotations have been added that confirm our predictions, including to the human protein MAP1LC3.

### Improvements to PANTHER trees

Seventeen fungal-specific autophagy-related proteins known to participate in autophagosome assembly did not belong to any PANTHER family at the time of annotation. The PANTHER family building process requires that sequences have a certain degree of similarity. Example include a number of *Schizosaccharomyces* pombe ATGs, specifically atg10, atg11, atg14 and atg20, with highly divergent sequences that failed to be included by the automatic PANTHER tree building process. After feedback from PAINT curators, the new version of PANTHER (v11) now includes these sequences in trees (in PTHR14957, PTHR13222, PTHR13664, PTHR10555, respectively). Moreover, the *S. cerevisiae* ATG11 gene used to be part of PTHR23160 (see Table 2). In the latest PANTHER version, ATG11 is correctly included in PTHR13222.

Also, some proteins relevant to autophagy are restricted to an extremely narrow clade of species. For example, *S. cerevisiae* Atg32p, a mitophagy-specific receptor that recruits the autophagic machinery to mitochondria and regulates selective degradation of mitochondria, only has orthologs in some yeasts and no orthologs in mammals (37). Most of these proteins that have limited phylogenic range are not yet part of any PANTHER family.

### Improvements to the ontology and experimental annotations

The process of reviewing the autophagic pathway revealed newly characterized autophagy-related proteins without any primary annotation to ‘autophagy’ or the more specific ‘autophagosome assembly’. The primary annotation of these proteins has been completed for further phylogenetic inference using PAINT. In other cases, there was no GO term available to describe a protein′s role. For example, the mammalian-specific protein ZFYVE1, a member of the PTHR22835 family, plays an important role in the early step of autophagosome formation and is used as a marker for omegasomes (38). However, since omegasomes have only been discovered recently, the GO term had not yet been created. We have completed the list of autophagosome formation-related GO terms by the creation 11 new GO component terms, including omegasome (GO:1990462) and terms describing the different possible membrane topologies for all autophagic vesicle proteins (Table 3). In addition to the creation of these new terms, we have also improved the name, definition, and hierarchy of 26 existing terms, and obsoleted 6 redundant terms. We have changed ‘autophagic vacuole’ to ‘autophagosome’ in 15 terms to be better aligned with the terminology used in the literature.

**Table 3.** GO-terms describing cellular components involved in autophagosome assembly

GO ID	Cellular component and corresponding topology
GO:0005776^a^	Autophagosome
GO:0000421^a^	Autophagosome membrane
GO:0034423^a^	Autophagosome lumen
GO:0097635^b^	Extrinsic component of autophagosome membrane
GO:0097636^b^	Intrinsic component of autophagosome membrane
^2^GO:0097637	Integral component of autophagosome membrane
GO:0000407	Pre-autophagosomal structure
GO:0034045	Pre-autophagosomal structure membrane
GO:0097632^b^	Extrinsic component of pre-autophagosomal structure membrane
GO:0097633^b^	Intrinsic component of pre-autophagosomal structure membrane
GO:0097634^b^	Integral component of pre-autophagosomal structure membrane
GO:1990462^b^	Omegasome
GO:1903349^b^	Omegasome membrane
GO:0097629^b^	Extrinsic component of omegasome membrane
GO:0097630^b^	Intrinsic component of omegasome membrane
GO:0097631^b^	Integral component of omegasome membrane

a Terms for which we have replaced ‘autophagic vacuole’ by ‘autophagosome’.

b Newly created terms.

### PAINT provides a coherent set of annotations

Primary annotations are derived from experiments done in various species that are models for different aspects of biology. Thus, those primary annotations can be rather heterogeneous. The strength of PAINT is that it involves strict selection of terms, which results in coherent annotations within proteins families, as well as across families implicated in a single process. Moreover, individual proteins may be annotated to a process but miss an annotation to the corresponding function or cellular component. Performing annotation at the scale of families rather than at the level of individual proteins increases the coherence of the annotations by selecting core functions of protein families as determined by inference among evolutionary neighbors.

#### Capturing main functions using PAINT

As described in the introduction, primary GO annotation is based on data from literature. In addition to the main roles of proteins, these annotations also include species-specific data, indirect roles such as upstream/downstream processes, as well as phenotypic descriptions. The PAINT annotation process involves reviewing all the primary GO annotations associated to a protein family, and selecting molecular functions and biological processes in which the family members play a direct role, and the cellular component where they take place.

An example of a phenotype annotation that has not been propagated to other species is the putative role of STK24 in the regulation of axon regeneration (39). Rat retinal ganglion cells lacking STK24 (Mst3b) failed to regenerate injured axons when stimulated by intraocular inflammation, while neuron-regenerating axons *in vivo* showed elevated STK24 activity, and reducing Mst3b expression attenuated regeneration and MAPK activation. While the primary annotation is not wrong when tagged as inferred by mutant phenotype, the data presented in the paper does not describe the molecular mechanism of STK24's role in axon regeneration, so it is not possible to determine whether the other proteins required for this process are present in other species. In these cases, annotations are not propagated.

Proteins involved in general membrane trafficking are another example of indirect roles being annotated (e.g. vesicular formation, transport, tethering and fusion). These are often also annotated to autophagy. We limited the PAINT inference of ‘autophagosome assembly’ to the proteins playing a specific direct role within the process being annotated. Families containing proteins that are involved in general membrane trafficking are therefore not annotated to ‘autophagosome assembly’.

#### PAINT as a quality assurance mechanism for primary GO annotation

A main concern of the GO consortium is to avoid over-annotation, which creates noise in the GO dataset. Non-relevant annotation of GO terms may be assigned based on indirect evidences such as phenotypes or due to incorrect use of GO terms that can, depending on context, both refer to an experimental assay and to a biological process. For many proteins, the only published data relating to function is limited to indirect phenotype after alteration of gene expression (deletion, over-expression) or mutagenesis. Six families contained proteins having annotations to ‘autophagosome assembly’, but lacked experimental evidence for a direct role in this process. This data was not used for annotation inference.

Similar over-annotations were observed for 49 families having members annotated to ‘execution phase of apoptosis’ (GO:0097194) or a child term. This led us to change some of the primary annotation to the more general term, ‘apoptotic process’ (GO:0006915). At the onset of the project in November 2013, there were 11 proteins with experimental annotations in human for ‘apoptotic DNA fragmentation’ (GO:0006309). After review, two annotations are left.

Most of these mis-annotations are due to capturing the indirect effects of deletions/mutations leading to apoptotic phenotypes such as DNA fragmentation. In these cases, the evidence points to a causal connection between a gene and apoptosis, but not a direct involvement in the execution of apoptosis itself.

Another source of false positive annotations is high throughput experiments (HTP), in particular subcellular localization experiments. These experiments have a relatively high level of false positives that can be attributed either to the methodology used for the purification processes, to the transient presence of proteins in the endoplasmic reticulum and in the Golgi apparatus during their biosynthesis, or in the vacuole when they are broken down. Note that for Phylogenetic Annotation we have selected the location(s) where the protein is functionally active, not transient locations. Moreover, high throughput methods are also subject to false positives based on the bioinformatics analysis method used. Usually a rate of 1% false discoveries is judged acceptable by journals; however as more and more data are integrated into GO, these false positives have an additive effect and the final rate of false positives may be much higher than 1%.

Primary, literature-based GO annotation requires expert knowledge of the protein and the process it is involved in. We favor using a process-based annotation, which produces annotations that are more consistent than single-paper-based annotation. The latter approach makes it very difficult for curators to select which term to use, since they have too little time to get familiar with the process they are annotating. Each phylogenetic tree, corresponding to a PANTHER family, takes between 1 and 10 h to annotate, thus approximately one full-time person for one month was needed for annotating the 36 families. This work confirms the usefulness of PAINT in rapidly assigning precise and biologically coherent annotations on large number of sequences.

#### Maintaining annotations up to date

As knowledge evolves, so do primary annotations. New processes are discovered, and new terms are created to describe them. Sometimes new knowledge requires obsoleting existing terms. PANTHER families are updated approximately yearly. At each update, the annotations in each tree are automatically reviewed to ensure that all supporting evidences are still available. Moreover, on rare occasions, clades can move from one tree to another, such as *S. cerevisiae* ATG11 (see ‘Improvements to PANTHER trees’ section). In this case, annotations are also migrated.

Trees are also monitored for other changes in the underlying data: obsoleted or merged GO terms, as well as removal of primary annotations, which may have a strong impact on PAINT annotation. When detected, curators review and update the annotations as required. These steps ensure that we maintain the annotations produced by PAINT up to date.

### Perspective

Gene Ontology annotations from the experimental literature have been accruing for over 15 years, and some genes have annotations to over a hundred distinct terms. This can make it challenging for users to distill the central roles of genes from within the sea of annotations. We find that many GO annotations are likely to be downstream effects of a more limited set of core biological ‘programs’ in which a gene directly participates. It is these core biological programs, and not the downstream effects, that the Phylogenetic Annotation process aims to identify. The gene sets for each biological program are more likely to display coherent expression under appropriate conditions, and thus we expect them to be useful for gene enrichment analysis.

On the other hand, it remains true that very few genes have been experimentally assayed for their functions, and even well characterized genes are missing direct experimental evidence for their most central roles. The GO Phylogenetic Annotation project also addresses this issue. A curator selects the most informative annotations among all homologs, and creates an explicit evolutionary model of how these functions arose. This model enables the consistent annotation of a large number of related genes, and can also be tested against future experimental annotations to identify how the model can be revised.

An important advantage that PAINT provides is a review of all primary annotations curated directly from the literature. The PAINT curator effectively reviews these primary annotations in the larger frame of an entire protein family, which gives a comprehensive overview of the current knowledge about a family and its evolution. This overview brings to the attention inconsistencies in primary annotations to PAINT curator. When appropriate, the PAINT curator reviews the experimental evidence, and based on this information resolve the inconsistencies using main functions for PAINT inference and leaving out the annotations that appear to be phenotypes or downstream effects. These reviewed annotations can be identified in the GO database simply by filtering for the IBA evidence code.

## Methods

PANTHER Version 9.0, containing genes from 85 completely sequenced genomes, was used for annotation. Annotation of models of functional evolution was done using PAINT version 1.13, which can be downloaded from Source Forge (http://sourceforge.net/projects/pantherdb/). All GO annotations are in the GO database (http://geneontology.org) and ancestral annotations are available from PanTree (http://pantree.org). PANTHER families, phylogenetic trees and multiple sequence alignments are available at http://pantherdb.org.
